# Childhood maltreatment and hair cortisol in pregnancy: role of recent stress

**DOI:** 10.1016/j.cpnec.2026.100389

**Published:** 2026-11-02

**Authors:** Michelle M.J. Mens, Chirag M. Vyas, Bohao Wu, Natalie Slopen, Shruthi Mahalingaiah, Jorge E. Chavarro, Rajarshi Mukherjee, Marc G. Weisskopf, Andrea L. Roberts

**Affiliations:** a Department of Social and Behavioral Sciences, Harvard T.H. Chan School of Public Health, Boston, MA, 02115, United States; b Department of Psychiatry, Massachusetts General Hospital, Boston, MA, 02114, United States; c Department of Environmental Health, Harvard T.H. Chan School of Public Health, Boston, MA, 02115, United States; d Department of Nutrition, Harvard T.H. Chan School of Public Health, Boston, MA, 02115, United States; e Department of Epidemiology, Harvard T.H. Chan School of Public Health, Boston, MA, 02115, United States; f Channing Division of Network Medicine, Department of Medicine, Brigham and Women's Hospital, Harvard Medical School, Boston, MA, 02115, United States; g Department of Biostatistics, Harvard T.H. Chan School of Public Health, Boston, MA, 02115, United States

**Keywords:** Childhood maltreatment, Cortisol, Stress, Insomnia, Pregnancy

## Abstract

Childhood maltreatment has been associated with long-lasting alterations in the hypothalamic-pituitary-adrenal axis, potentially reflected in cortisol dysregulation, and with increased risk of adverse pregnancy and offspring developmental outcomes. Yet, it remains unclear whether childhood maltreatment and indicators of recent stress are associated with hair cortisol during pregnancy and whether recent stress modifies the association between childhood maltreatment and cortisol during pregnancy. To address these knowledge gaps, we collected hair samples from 148 pregnant women in the Nurses’ Health Study 3. Hair cortisol was measured from 3-cm samples (mean gestational age: 18.3 weeks [range: 9–33]). Childhood maltreatment was ascertained using the validated 28-item Childhood Trauma Questionnaire (CTQ), measuring five abuse and neglect subscales. Participants also reported indicators of recent stress (perceived stress, depressive symptoms, anxiety, past-year stressful life events, and insomnia). Multivariable linear regression models were used for all analyses. In primary analyses, childhood maltreatment was not significantly associated with hair cortisol levels. Among indicators of recent stress, past-year stressful life events — but not other indicators — were significantly associated with lower hair cortisol levels. In secondary analyses, no significant interactions were observed between childhood maltreatment and indicators of recent stress. In an exploratory analysis, among women with moderate-to-severe childhood maltreatment, high insomnia symptoms (top 20%) were associated with lower hair cortisol levels. This exploratory finding suggests that insomnia symptoms may be relevant to understanding cortisol dysregulation among pregnant women with a history of childhood maltreatment, though the direction and mechanisms of this association warrant further investigation in larger samples.

## Highlights

• Unclear if childhood maltreatment and recent stress relate to pregnancy cortisol.
• In 148 pregnant women, childhood maltreatment was not significantly associated with hair cortisol.
• Past-year stressful life events, but not other stress indicators, were associated with lower hair cortisol levels.
• In exploratory analyses, insomnia symptoms modified the maltreatment-hair cortisol association.
• Larger, more diverse studies should examine maltreatment, sleep disturbance, and hair cortisol during pregnancy.

## 1 Introduction

Childhood maltreatment – including emotional, physical, and sexual abuse, as well as emotional and physical neglect – is highly prevalent and has enduring consequences for physical and mental health [1]. In the United States, approximately one in seven children experiences maltreatment [2], conferring elevated risks for cardiometabolic disease [1], [3], [4], depression [5], [6], and post-traumatic stress disorder (PTSD) [7], [8]. Emerging evidence suggests that these adverse consequences of childhood maltreatment may extend across generations, with offspring of exposed mothers having an increased likelihood of cardiometabolic and neurodevelopmental impairments [9], [10], [11], [12], [13], [14], [15].

One potential mechanistic pathway linking early adversity to offspring adverse health outcomes is dysregulation of the hypothalamic-pituitary-adrenal (HPA) axis during gestation. Childhood maltreatment has been associated with both heightened and blunted cortisol responses in adulthood [16], [17]. During pregnancy, cortisol levels rise progressively, increasing two-to four-fold compared to non-pregnancy levels, driven by the release of estrogen and placental corticotropin-releasing hormone that interact with the maternal HPA axis to stimulate cortisol secretion [18], [19], [20]. Given these substantial pregnancy-induced alterations in HPA axis regulation, associations between childhood maltreatment and cortisol during pregnancy may differ from those observed in non-pregnant individuals [21]. Because cortisol plays a critical role in fetal maturation, maternal cortisol dysregulation during pregnancy may affect offspring health [16], [22].

Despite growing interest, evidence on the association between childhood maltreatment and cortisol during pregnancy is limited [17], [23], [24]. Most prior studies have relied on salivary or serum cortisol, which capture short-term fluctuations [17]. A systematic review and meta-analysis of 87 studies found evidence of blunted cortisol stress reactivity following childhood maltreatment but no overall differences in basal cortisol measures, suggesting that associations may depend on the type of cortisol measure assessed [24]. Hair cortisol concentration, by contrast, provides an integrated, long-term biomarker of cumulative cortisol secretion over several months [25]. Among studies using this measure, a meta-analysis of 28 studies (n = 3397) on childhood adversity and hair cortisol suggested small and mixed associations among young adults [17], while a recent large study of pregnant women (n = 1822) found modestly elevated hair cortisol among those exposed to physical or sexual abuse [23]. In contrast, several other studies during pregnancy have reported no associations between childhood maltreatment and hair cortisol [26], [27], [28], further highlighting the heterogeneous nature of the existing evidence. Notably, neglect - the most prevalent form of maltreatment - has been understudied in this context [29]; to the best of our knowledge, only one small study (n = 51) has examined neglect in relation to hair cortisol during pregnancy, reporting lower third-trimester levels [30]. Moreover, given that stronger associations between HPA-axis activity and adverse offspring health outcomes have been documented earlier in pregnancy [31], studies restricted to the third trimester may fail to capture the most critical window.

Taken together, the existing literature does not provide a sufficiently consistent basis for predicting the direction of associations between childhood maltreatment and hair cortisol. Both heightened and blunted cortisol activity have been reported following childhood adversity, and findings may vary according to characteristics of the adversity and the developmental and physiological context in which HPA-axis activity is assessed [16], [17]. This uncertainty may be particularly relevant during pregnancy given the substantial physiological changes in HPA-axis activity described above. Accordingly, the study was guided by specific research questions, without a priori hypotheses regarding the direction of associations with hair cortisol.

The stress-sensitization theory suggests that early adversity recalibrates stress-response systems, heightening vulnerability to subsequent psychological distress across the lifespan [32], [33]. Consistent with this, women with histories of childhood maltreatment are more likely to experience elevated levels of stress during pregnancy [34], yet no prior research has examined whether recent stress modifies the association between childhood maltreatment and hair cortisol. Recent stress and stress-related functioning can be characterized across distinct domains. Stressful life events capture exposure to recent external stressors, perceived stress reflects subjective stress appraisal, and depressive, anxiety, and insomnia symptoms capture psychological and behavioral correlates of stress. These domains have been associated with HPA-axis functioning, although their associations with cortisol are heterogeneous, particularly during pregnancy [16], [35], [36]. We therefore examined these indicators separately rather than treating them as interchangeable measures of a single recent-stress construct. Stress-sensitization theory further suggests that associations between childhood maltreatment and HPA-axis functioning may differ according to the level of recent stress or stress-related symptoms. Given the limited evidence regarding such effect modification during pregnancy, we additionally explored whether the childhood maltreatment–hair cortisol association differed between participants with low versus elevated indicators of recent stress.

Therefore, in a sample of predominantly second-trimester pregnant women from the Nurses’ Health Study 3 (NHS3) [37], we analyzed 3-cm hair samples reflecting three months of cumulative cortisol secretion. Our primary objectives were: 1) to examine the association between childhood maltreatment and hair cortisol; and 2) to assess the independent associations between indicators of recent stress (perceived stress, depressive symptoms, anxiety, stressful life events, and insomnia) and hair cortisol. Secondarily, we examined associations between specific maltreatment subtypes and hair cortisol, and further examined whether indicators of recent stress modified the childhood maltreatment–hair cortisol association. Guided by stress-sensitization theory, we additionally conducted exploratory analyses examining effect modification by elevated indicators of recent stress.

## 2 Methods

### 2.1 Sample

The Nurses' Health Study 3 (NHS3) is an ongoing longitudinal cohort initiated in 2010, enrolling U.S. and Canadian nurses and nursing students born after January 1, 1965. By January 2023, 36,010 had completed one or more follow-up questionnaires and were considered participants [37]. Participants are surveyed biannually on lifestyle, reproductive health, and occupational exposures. In October 2020, the Maternal Health, Maternal Biology sub-study began, examining how childhood maltreatment affects biological markers during pregnancy. NHS3 women planning or currently pregnant were asked to join; eligible participants under 24 weeks gestation were invited to donate hair samples. Women with twin pregnancies were not eligible for the study because hormonal profiles differ substantially between twin and singleton pregnancies, which could affect hair cortisol concentrations. Participants residing outside the United States were not invited to provide biospecimens because of logistical challenges associated with international biosample shipment. Of 430 pregnant enrollees, 365 agreed to donate hair, with 158 returning samples (43.3% return rate) by July 29, 2024. Participants were reminded via email at multiple time points to collect and return their samples. Informed written consent was provided by participants at sub-study enrolled. Study protocols were approved by the Institutional Review Board of the Mass General Brigham (protocol #2020P001020). This study was not pre-registered. At the time data collection commenced in this sub-study, pre-registration was not yet a standard requirement in this field of research.

### 2.2 Ascertainment of childhood maltreatment

Pregnant women who consented to participate in the MHMB sub-study were asked to complete a questionnaire in which childhood maltreatment was assessed using the Childhood Trauma Questionnaire (CTQ) [38], [39]. The CTQ consists of 28 items with statements beginning with the phrase, “When I was growing up, during my 18 years of life …”. Out of 28 CTQ items, 25 measure childhood maltreatment, including 5 abuse and neglect subscales (physical abuse, emotional abuse, sexual abuse, physical neglect, and emotional neglect), each consisting of 5 items respectively. The remaining 3 CTQ items are designed to measure Minimization/Denial (M/D). Since the validity of these items has not been established in the literature, we did not include them in the computation of the CTQ score [40].

The response options for all 5 abuse and neglect subscales range from “never true” (1 point) to “very often true” (5 points). Responses to positive items—such as whether the respondent felt loved, whether they were made to feel important, and whether they were looked out for—were reverse-coded so that “never true” corresponded to 5 points and “very often true” corresponded to 1 point. Therefore, each subscale can range from 5 to 25 points. A total CTQ score summed all responses (range: 25-125 points); higher scores indicated more severe experiences (Cronbach's alpha = 0.77). Leveraging prior work [41], [42], we categorized a total CTQ score into four groups: 25 points as no maltreatment (reference group), 26-29 points as “very low maltreatment,” 30-36 points as “low maltreatment,” and >36 points as “moderate to severe maltreatment.” Participants missing more than 16 items were excluded (*n* = 1). Continuous scores for each maltreatment type were calculated [39], [43], [44]. The continuous CTQ total score was used in the primary analysis, while continuous CTQ subscale scores were used in the secondary analyses examining specific maltreatment types. The four CTQ severity categories (no, very low, low, and moderate-to-severe maltreatment) were used to characterize the distribution of childhood maltreatment descriptively. For the exploratory effect-modification analyses, participants in the no, very low, and low maltreatment categories were combined to form a none-to-low maltreatment reference group, which was compared with participants with moderate-to-severe maltreatment. Cronbach's alphas were: emotional abuse = 0.88, physical abuse = 0.56, sexual abuse = 0.93, emotional neglect = 0.89, and physical neglect = 0.75.

### 2.3 Indicators of recent stress

Indicators of recent stress were analyzed as continuous scores to maximize statistical power. However, because the “stress sensitization” literature suggests that adverse health outcomes may be most pronounced at high levels of both childhood maltreatment and recent stress [45], [46], [47], we conducted exploratory analyses in which stress indicators were dichotomized into low/moderate versus high levels. Where established clinical cutoffs were available, these were used to define high stress; for measures without validated cutoffs, distribution-based thresholds were applied as described below.

#### 2.3.1 Perceived stress

Past-month perceived stress was measured using the 4-item Perceived Stress Scale (PSS-4) [48]. Participants rated items such as, “How often have you felt that things were going your way?” Responses ranged from “never” (1) to “very often” (4), with positive items reverse-coded. Higher total scores indicated greater perceived stress (Cronbach's alpha = 0.79). For exploratory analyses, scores ≥6 points were used to indicate high perceived stress [49].

#### 2.3.2 Depressive symptoms

Past-week depressive symptoms were measured using the 10-item Center for Epidemiological Studies-Depression (CES-D-10) scale [50], [51]. Responses ranged from ‘rarely or none of the time’ (1) to “all the time” (4), with positive items reverse-coded (Cronbach's alpha = 0.70). For exploratory analysis, total scores ≥10 points were used to indicate significant depressive symptoms [52].

#### 2.3.3 Anxiety symptoms

Anxiety symptoms were evaluated using the 2-item Generalized Anxiety Disorder questionnaire (GAD-2), asking about experiences in the past two weeks (e.g., “I was bothered by not being able to stop or control worrying”) [53]. Responses ranged from “not at all” (1) to “nearly every day” (4) (Cronbach's alpha = 0.67). For exploratory analysis, scores ≥3 points were used to indicate possible generalized anxiety disorder [54].

#### 2.3.4 Past-year stressful events

Participants reported stressful life events over the past year using 15 items from the Alcohol Use Disorder and Associated Disabilities Interview Schedule-IV (AUDADIS) [55]. Events included economic stressors (e.g., “Were you fired or laid off?”), interpersonal stressors (e.g., “Did you get divorced?”), crime-related stressors, and other stressors (e.g., serious illness or injury). The total number of past-year events was summed to create a continuous score for primary and secondary analyses. For exploratory analyses, participants were dichotomized into those reporting any versus no stressful life events.

#### 2.3.5 Insomnia

Past-month insomnia was assessed with the 5-item Women's Health Initiative Insomnia Rating Scale (WHIIRS; [56]; e.g., “Did you have trouble falling asleep?”). Responses ranged from “Not in the past 4 weeks” (1) to “Five or more times a week” (4), with scores summed (Cronbach's alpha = 0.81). The continuous WHIIRS score was used in primary and secondary analyses. For exploratory analyses, given the absence of a validated clinical cutoff, participants scoring in the top 20% (WHIIRS ≥12) were classified as having high insomnia symptoms.

### 2.4 Covariate selection

To reduce unexplained variance, we considered covariates based on previous literature [57], [58], [59], [60]: age at hair collection, racial identity (White, Black, Asian, Hispanic, Other), gestational age at hair collection (in weeks), use of hair treatment (coloring, bleaching, straightening), hair washing in the last 24 h, typical exercise>2h/day, swimming in a chlorinated pool (≥1/week for last 3 months), cortisol medication use (topical, oral, or nasal), medical conditions and their medication use, including diabetes mellitus, thyroid disorders (e.g., hypothyroidism or hyperthyroidism), and other dysregulations of the HPA axis (e.g., Cushing's disease or Addison's disease). Due to the relatively small sample size, we retained only covariates associated with hair cortisol in our data (see Supplementary Methods). We retained as covariates gestational age (weeks), frequent exercise (>2h/day) and/or swimming in a chlorinated pool (≥1/week, past 3 months), and cortisol medication use (topical, oral, or nasal). Additionally, assay batch was included as a covariate to account for potential batch effects.

### 2.5 Hair cortisol concentration assessment

Participants collected hair samples at home using standardized written instructions and collection materials provided by the study. Participants were instructed to separate a bundle of hair approximately the thickness of a pencil from the back of the head, secure it with the provided elastic band close to the scalp, and cut the hair as close to the scalp as possible without removing the roots. Samples were required to be at least 3 cm in length. Participants placed the collected hair in a sealed plastic bag and were instructed to store the sample in a dry, dark place until shipment. Participants also completed a hair questionnaire at the time of collection. Samples were returned to the Channing Laboratory of Mass General Brigham by regular USPS first-class mail using a provided pre-stamped padded envelope. Upon receipt, hair samples were subsequently transported by FedEx to the Dettmer Lab at Yale University for cortisol analysis. Technicians measured hair length to the nearest millimeter and prepared a 3-cm segment proximal to the scalp, reflecting approximately three months of cumulative cortisol secretion. Samples were weighed, washed twice with isopropanol, air-dried, and ground into powder, then stored at −80°C. For cortisol extraction, methanol was added, and samples were incubated overnight. The extracts were aliquoted, dried in a SpeedVac for at least 90 min, and reconstituted with 0.25 mL of assay buffer. Because hair grows approximately 1 cm per month, each 1-cm portion of the analyzed hair segment reflects approximately the preceding month of cortisol accumulation. Consequently, trimester at hair collection does not necessarily correspond exclusively to the trimester represented by the hair cortisol concentration measurement; samples collected early or midway through the second trimester may partly reflect first-trimester cortisol exposure.

Cortisol concentrations were measured using the Cortisol Enzyme Immunoassay kit, with a lower limit of detection (LLOD) of 0.45 pg/mg and adjusted for hair weight. Assessments were performed in two batches (first batch intra-assay CV = 9.0%; second batch CV = 23.6%). Hair cortisol concentrations were log-transformed prior to all analyses to address their non-normal distribution. We excluded samples weighing less than 10 mg due to unreliable readings (n = 5), samples with high coefficients of variation in repeated assays (n = 2), and participants with incomplete questionnaires (n = 2), yielding a final analytic sample of 148 predominantly second-trimester pregnant women.

### 2.6 Missing data

For participants with hair cortisol levels below the lower limit of detection (n = 9), we imputed missing values at 0.40 pg/mg (10% below the lower limit of detection of 0.45 pg/mg) to maintain rank order and avoid overestimation [61], [62]. Eight participants were missing birth date, preventing age calculation (5.4%); these ages were imputed with the mean age, 34.0 years. For nine samples missing gestational age (6.1%), the mean, 18.3 weeks, was used. Missing CTQ item-level data were handled using predictive mean matching (PMM), implemented with the mice package in R. Individuals with more than 16 missing CTQ items were excluded from the analysis (n = 1). For individuals with 1 to 15 missing items (n = 3), missing responses were imputed at the item level. Five imputed datasets were generated using 50 iterations and random seed = 500 with the R package “mice” [63].

## 3 Statistical analysis

### 3.1 Post-hoc power analyses

Given that our original power calculations were based on higher anticipated enrollment, we performed post-hoc power analyses using a non-central F-distribution approach, based on our primary regression model (N = 148, five predictors including covariates, alpha = 0.05, two-tailed). Our study had 21% power to detect a small effect (f^2^ = 0.02), 51% power to detect a small-to-medium effect (f^2^ = 0.05), and 97% power to detect a medium effect (f^2^ = 0.15). The minimum detectable effect size at 80% power was f^2^ = 0.09, corresponding to a small-to-medium effect. These estimates indicate that the study was adequately powered to detect medium-to-large effects only and was likely underpowered to detect the small-to-moderate effects.

### 3.2 Descriptive statistics and correlations

Participants' characteristics [mean (SD) or n (%)] were reported by childhood maltreatment exposure (none, very low, low, moderate-severe). Group differences were assessed using chi-square tests for categorical variables and Student's t-tests for continuous variables. Log-transformed hair cortisol levels were also reported across childhood maltreatment exposure categories. Spearman correlations were calculated between childhood maltreatment and indicators of recent stress to characterize their interrelationships prior to regression analyses, given non-normal distributions of study variables.

### 3.3 Primary analyses

We examined the association between childhood maltreatment and hair cortisol concentration using multivariable linear regression models with the CTQ total score as the primary independent variable, modeled continuously. To assess the independent associations between indicators of recent stress and hair cortisol, we fit separate multivariable linear regression models for each continuous indicator of recent stress (perceived stress, depressive symptoms, anxiety, stressful life events, and insomnia). All models were adjusted for gestational age at hair sampling (weeks), batch, exercise/swimming, and topical/oral/nasal cortisol medication use. Hair cortisol was log-transformed prior to all analyses to address right skewness. Regression model assumptions were evaluated through inspection of residual distributions and Q–Q plots and using the Shapiro–Wilk test for residual normality. Statistical analyses were conducted in R using the lm() function. Continuous predictors were entered into their original measurement units and were not z-scored before model estimation. Unstandardized regression coefficients (B) with 95% confidence intervals are reported for all models. For the primary analyses, standardized regression coefficients (β) were additionally calculated to facilitate comparison of effect sizes across continuous predictors measured on different scales. Standardized coefficients were calculated for reporting purposes; the regression models themselves were fitted using predictors in their original measurement units. Because these indicators were treated as conceptually distinct exposures and each association with hair cortisol was evaluated separately, no multiple-comparison correction was applied across these models; however, the number of associations examined was considered when interpreting the findings. Statistical significance was therefore defined as p < 0.05, and 95% confidence intervals were calculated for all estimates.

### 3.4 Secondary analyses

To examine associations between specific maltreatment subtypes and hair cortisol, we fit separate linear regression models for each of the five CTQ subscales (emotional abuse, physical abuse, sexual abuse, emotional neglect, physical neglect) as continuous independent variables. All models were adjusted for gestational age at hair sampling (weeks), assay batch, exercise/swimming, and topical/oral/nasal cortisol medication use Standardized regression coefficients (β) were additionally reported for the CTQ subscale analyses to facilitate comparison of effect sizes across the continuous maltreatment subscales.

To examine whether indicators of recent stress modified the childhood maltreatment–hair cortisol association, we fit separate multivariable linear regression models including an interaction between the continuous CTQ total score and each continuous indicator of recent stress. Interaction terms were specified in R using the * operator (e.g., lm(log_hair_cortisol ∼ CTQ_total * recent_stress + covariates [gestational age at hair sampling, assay batch, exercise/swimming, and topical/oral/nasal cortisol medication use])), which includes both main effects and their product term. The continuous predictors were not z-scored before calculation of the interaction terms; therefore, the models were estimated using predictors and interaction terms in their original measurement units. The significance of each interaction term was evaluated using its regression coefficient, 95% confidence interval, and p-value. Given the multiple comparisons inherent in testing five CTQ subscales and five interaction models separately, Bonferroni correction was applied within each set of secondary analyses, with a corrected significance threshold of p < 0.010 (0.05/5).

### 3.5 Exploratory analyses

In an exploratory analysis, we examined whether dichotomized indicators of recent stress modified the childhood maltreatment–hair cortisol association. Participants in the no, very low, and low maltreatment categories were combined to form the none-to-low maltreatment reference group and compared with participants with moderate-to-severe maltreatment. We tested interactions between childhood maltreatment group (none-to-low vs. moderate-to-severe) and each recent stress indicator (perceived stress, depressive symptoms, anxiety, stressful life events, and insomnia), classified as low versus high using the thresholds described above. Separate linear regression models were fit for each interaction and adjusted for gestational age at hair sampling (weeks), assay batch, exercise/swimming, and topical/oral/nasal cortisol medication use.

### 3.6 Sensitivity analyses

To examine potential confounding by gestational timing, we conducted sensitivity analyses comparing hair cortisol levels and the prevalence of indicators of recent stress across trimesters. Given the wide range of gestational age at hair sample collection (9–33 weeks), these analyses were conducted to assess whether trimester of collection influenced the primary results. Additionally, we reran all primary models (associations between childhood maltreatment, recent stress indicators, and log hair cortisol) restricted to participants in the second trimester given that most hair cortisol samples were collected during this period. Because each 1 cm of the analyzed hair segment represents approximately one month of cortisol accumulation, samples collected during the early second trimester may partly reflect first-trimester cortisol exposure. We therefore conducted an additional sensitivity analysis assuming an approximate four-week hair cortisol concentration exposure window. This analysis was restricted to participants whose estimated four-week window fell entirely within the second trimester, corresponding to hair collection at gestational weeks 18–27. To assess whether the substitution of hair cortisol values below the assay LOD influenced the findings, we conducted an additional sensitivity analysis excluding participants with hair cortisol values below the LOD (0.45 pg/mg) and repeated all primary regression models.

All analyses were conducted using R (Version 4.2.0; [64]). Linear regression models were fit using the *lm(****)*** function in R. Data management was conducted using the dplyr, tidyr, and purrr packages [65], [66], [67], and hair cortisol and stress-related variables were summarized using psych [68]. Assumption checks and normality tests (e.g., Shapiro–Wilk) were conducted with rstatix [69]. Correlation matrices and heatmaps were generated using ggcorrplot (with Spearman's rank correlation; [70]), and additional data visualizations were created with ggplot2 [71].

## 4 Results

### 4.1 Descriptive statistics and correlations

Characteristics of the 148 participants are in Table 1. Among them, 28 (18.9%) experienced no childhood maltreatment, 42 (28.4%) had very low, 43 (29.1%) had low, and 35 (23.6%) had moderate to severe maltreatment. Additionally, 28 (18.9%) had high perceived stress, 25 (16.9%) had high depressive symptoms, 13 (8.8%) had high anxiety, and 28 (18.9%) experienced three or more stressful life events in the past year, with 3 (2.0%) having two events in the past month. Twenty-seven (18.2%) had high insomnia. Log-transformed hair cortisol levels were similar across childhood maltreatment exposure groups (Supplementary Table 2).

**Table 1:** Characteristics of pregnant women of the NHS3 participants included in this study (n = 148).

	Overall	No maltreatment (CTQ score = 25)	Low maltreatment (CTQ score = 26-29)	Medium maltreatment (CTQ score = 30-36)	High maltreatment (CTQ score >36)	p- value
n	148	28	42	43	35	
Age, years (mean (SD))	33.53 (3.46)	33.77 (3.19)	32.81 (3.50)	33.45 (3.81)	34.29 (3.10)	0.30
Race (%)						0.32
*Asian*	3 (2.0)	0 (0.0)	1 (2.4)	0 (0.0)	2 (5.7)	
*Black*	3 (2.0)	0 (0.0)	1 (2.4)	2 (4.7)	0 (0.0)	
*Hispanic*	1 (0.7)	0 (0.0)	0 (0.0)	0 (0.0)	1 (2.9)	
*Other*	8 (5.4)	1 (3.6)	2 (4.8)	1 (2.3)	4 (11.4)	
*White*	133 (89.9)	27 (96.4)	38 (90.5)	40 (93.0)	28 (80.0)	
Batch (batch = 2) (%)	87 (58.8)	17 (60.7)	25 (59.5)	23 (53.5)	22 (62.9)	0.85
Gestational age, weeks (range 9-33) (mean (SD))	18.27 (6.34)	17.37 (6.59)	18.72 (6.48)	18.55 (6.11)	18.12 (6.44)	0.83
Medical condition (%)	18 (12.2)	1 (3.6)	8 (19.0)	3 (7.0)	6 (17.1)	0.13
*Mental health (%)*	13 (8.8)	0 (0.0)	6 (14.3)	3 (7.0)	4 (11.4)	0.19
*Thyroid (%)*	5 (3.4)	1 (3.6)	2 (4.8)	0 (0.0)	2 (5.7)	0.51
Exercise (%)	1 (0.7)	0 (0.0)	0 (0.0)	0 (0.0)	1 (2.9)	0.36
Swimming in pool (%)	13 (8.8)	3 (10.7)	3 (7.1)	3 (7.0)	4 (11.4)	0.86
Hair treatment (%)	27 (18.2)	5 (17.9)	9 (21.4)	7 (16.3)	6 (17.1)	0.93
*Bleach (%)*	5 (3.4)	1 (3.6)	2 (4.8)	2 (4.7)	0 (0.0)	0.64
*Coloring (%)*	23 (15.5)	5 (17.9)	7 (16.7)	6 (14.0)	5 (14.3)	0.96
*Straightening (%)*	2 (1.4)	0 (0.0)	1 (2.4)	0 (0.0)	1 (2.9)	0.59
Nasal cortisol use (%)	16 (10.8)	1 (3.6)	8 (19.0)	3 (7.0)	4 (11.4)	0.16
Topical or oral cortisol use (%)	11 (7.4)	2 (7.1)	4 (9.5)	3 (7.0)	2 (5.7)	0.93
High perceived stress (%)	28 (18.9)	2 (7.1)	5 (11.9)	12 (27.9)	9 (25.7)	0.07
High depressive symptoms (%)	25 (16.9)	3 (10.7)	3 (7.1)	10 (23.3)	9 (25.7)	0.08
High anxiety symptoms (%)	13 (8.8)	3 (10.7)	3 (7.1)	4 (9.3)	3 (8.6)	0.96
Stressful life events past year (%)						0.05
0	40 (27.0)	5 (17.9)	18 (42.9)	11 (25.6)	6 (17.1)	
1	56 (37.8)	16 (57.1)	10 (23.8)	19 (44.2)	11 (31.4)	
2	24 (16.2)	2 (7.1)	10 (23.8)	7 (16.3)	5 (14.3)	
3	19 (12.8)	3 (10.7)	4 (9.5)	4 (9.3)	8 (22.9)	
4	4 (2.7)	1 (3.6)	0 (0.0)	1 (2.3)	2 (5.7)	
5	5 (3.4)	1 (3.6)	0 (0.0)	1 (2.3)	3 (8.6)	
Stressful life events past month (%)						0.64
0	110 (74.3)	19 (67.9)	31 (73.8)	32 (74.4)	28 (80.0)	
1	35 (23.6)	9 (32.1)	9 (21.4)	10 (23.3)	7 (20.0)	
2	3 (2.0)	0 (0.0)	2 (4.8)	1 (2.3)	0 (0.0)	
High insomnia (%)	27 (18.2)	5 (17.9)	6 (14.3)	7 (16.3)	9 (25.7)	0.60

**Note.** Values are presented as *n* (%) unless otherwise indicated. CTQ = Childhood Trauma Questionnaire; SD = standard deviation. CTQ severity categories were defined as no maltreatment (CTQ = 25), very low maltreatment (CTQ = 26–29), low maltreatment (CTQ = 30–36), and moderate-to-severe maltreatment (CTQ > 36). High perceived stress was defined as PSS-4 ≥ 6; high depressive symptoms as CES-D-10 ≥ 10; high anxiety symptoms as GAD-2 ≥ 3; and high insomnia symptoms as WHIIRS ≥ 12 (top 20% of the distribution). *p*-values represent comparisons across the four childhood maltreatment groups.

Fig. 1 illustrates the correlations between CTQ subscales and recent distress and stressors. In this sample, emotional abuse and neglect were highly correlated (r = 0.78), as were physical and emotional neglect (r = 0.68). Furthermore, perceived stress and depressive symptoms showed a modest correlation (r = 0.61), followed by depressive symptoms and anxiety (r = 0.57), and perceived stress and anxiety (r = 0.55).

**Fig. 1:**
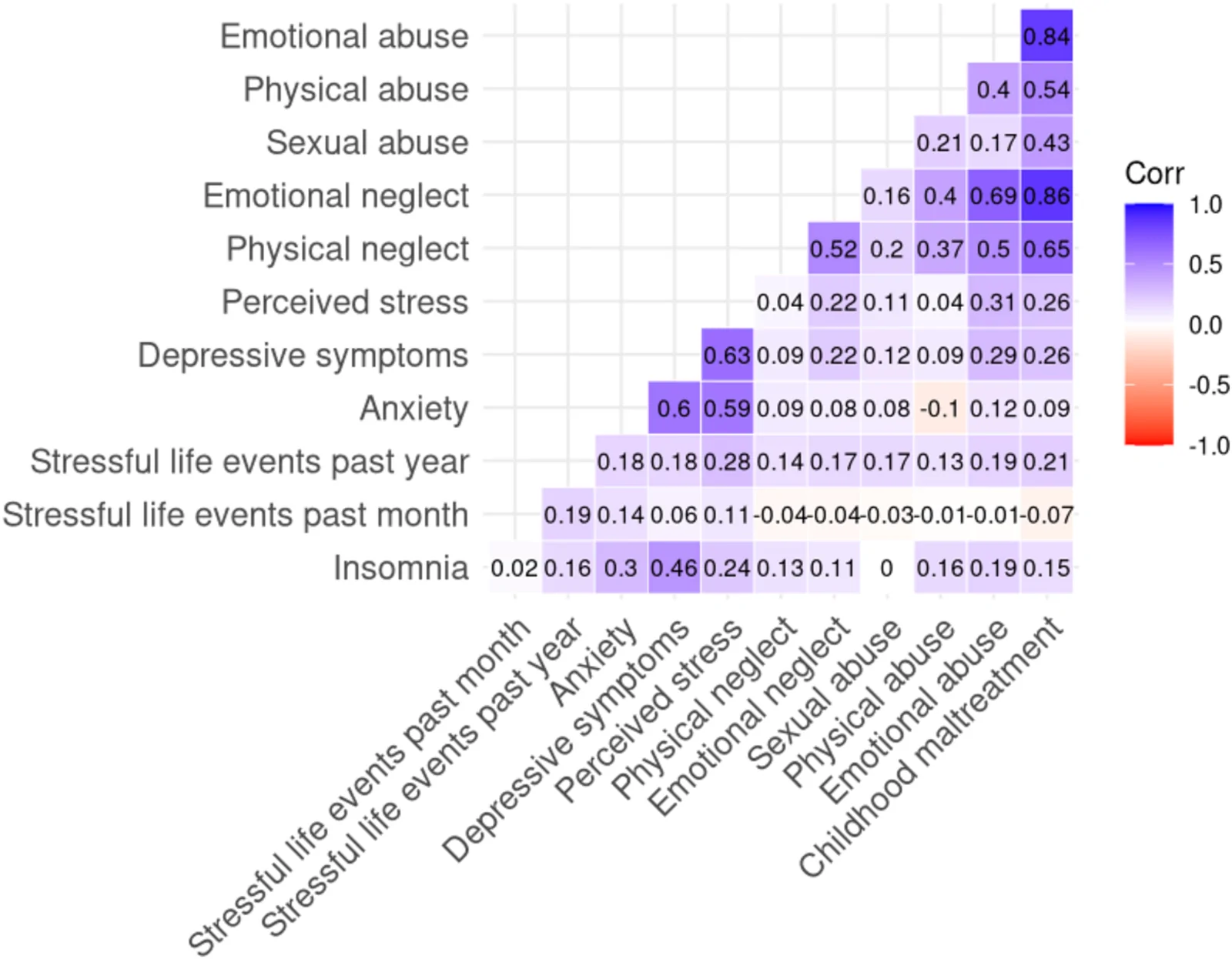
Spearman correlation matrix depicting associations between childhood maltreatment (CTQ total and subscales: emotional abuse, physical abuse, sexual abuse, emotional neglect, physical neglect) and indicators of recent psychological distress and stressors (perceived stress, depressive symptoms, anxiety symptoms, past-year and past-month stressful life events, and insomnia symptoms) in pregnant women from the NHS3 (n = 148). Correlations are Spearman's rank coefficients (ρ) based on pairwise complete observations.

### 4.2 Primary analyses

Childhood maltreatment, assessed as the continuous CTQ total score, was not associated with hair cortisol concentration (B = 0.003, β = 0.03, 95% CI: −0.014 to 0.019, p = 0.74). Among continuous indicators of recent stress, past-year stressful life events — but not perceived stress, depressive symptoms, anxiety symptoms, or insomnia — were significantly associated with lower hair cortisol levels (B = −0.13, β = −0.17, 95% CI: −0.263, −0.006, p = 0.04, Table 2). Complete estimates from the primary regression models, including coefficients, standard errors, 95% confidence intervals, and p-values for all adjustment covariates, are reported in Supplementary Table 3.

**Table 2:** Results of the estimated associations between indicators of recent stress and hair cortisol levels in pregnant women (n = 148).

Indicator of recent stress	B	β	SE	95% CI	p-value
Depressive symptoms	0.007	0.024	0.022	−0.038, 0.051	0.770
Anxiety symptoms	−0.053	−0.057	0.078	−0.206, 0.100	0.496
Perceived stress	0.014	0.035	0.035	−0.054, 0.083	0.680
Life events, past month	0.010	0.005	0.171	−0.328, 0.347	0.954
Life events, past year	−0.135	−0.172	0.065	−0.263, −0.006	**0.040**
Insomnia symptoms	0.001	0.004	0.020	−0.038, 0.040	0.960

**Note.** B = unstandardized regression coefficient; β = standardized regression coefficient; SE = standard error; CI = confidence interval. B represents the expected change in log-transformed hair cortisol associated with a one-unit increase in the corresponding recent stress indicator. Standardized β represents the association expressed in standard deviation units and is provided to facilitate comparison of effect sizes across indicators measured on different scales. Estimates are from separate multivariable linear regression models for each recent stress indicator, adjusted for assay batch, gestational age at hair sampling (weeks), exercise/swimming, and topical, oral, or nasal cortisol use. Statistical significance was defined as *p* < 0.05; statistically significant associations are shown in bold.

### 4.3 Secondary analyses

No significant associations between hair cortisol and any individual CTQ subscale remained after Bonferroni correction (Supplementary Table 4). Similarly, interaction terms involving childhood maltreatment and recent stress indicators were not statistically significant (Supplementary Table 5).

### 4.4 Exploratory analyses

In an exploratory analysis using dichotomized variables, a significant interaction was observed between moderate-to-severe childhood maltreatment and high insomnia symptoms (β = −0.95, p = 0.04; Table 3), indicating that among women with moderate-to-severe childhood maltreatment, high insomnia symptoms (top 20%) were associated with lower hair cortisol levels.

**Table 3:** Effect modification by binary indicators of recent stress in the association between severity of childhood maltreatment and log hair cortisol in pregnant women (n = 148).

Effect modifier (binary recent stress indicator)	B (interaction)	SE	95% CI	p-value
Severe maltreatment × high depressive symptoms	−0.107	0.48	−1.057, 0.843	0.824
Severe maltreatment × high anxiety symptoms	−1.088	0.70	−2.464, 0.288	0.120
Severe maltreatment × high perceived stress	−0.367	0.48	−1.310, 0.575	0.442
Severe maltreatment × high life events, past month	0.132	0.47	−0.800, 1.064	0.780
Severe maltreatment × high life events, past year	0.011	0.14	−0.269, 0.292	0.936
Severe maltreatment × insomnia	−0.946	0.46	−1.863, −0.029	**0.043**

**Note.** B = unstandardized regression coefficient; SE = standard error; CI = confidence interval; CTQ = Childhood Trauma Questionnaire; PSS-4 = 4-item Perceived Stress Scale; CES-D-10 = 10-item Center for Epidemiological Studies Depression Scale; GAD-2 = 2-item Generalized Anxiety Disorder scale; AUDADIS = Alcohol Use Disorder and Associated Disabilities Interview Schedule; WHIIRS = Women's Health Initiative Insomnia Rating Scale. B (interaction) represents the unstandardized regression coefficient for the product term between childhood maltreatment group and each binary recent stress indicator. Participants with no, very low, or low maltreatment were combined to form the none-to-low maltreatment reference group and compared with participants with moderate-to-severe maltreatment. High perceived stress was defined as PSS-4 ≥ 6, high depressive symptoms as CES-D-10 ≥ 10, high anxiety symptoms as GAD-2 ≥ 3, and high insomnia symptoms as WHIIRS ≥ 12 (top 20% of the distribution). Stressful life events were classified as described in the Methods. Estimates are from separate multivariable linear regression models for each effect modifier, adjusted for assay batch, gestational age at hair sampling (weeks), exercise/swimming, and topical, oral, or nasal corticosteroid use. These analyses were exploratory and should be interpreted as hypothesis-generating. Statistical significance was defined as p < 0.05; statistically significant interaction terms are shown in bold.

### 4.5 Sensitivity analyses

Hair cortisol was slightly higher in women who donated hair in later versus earlier trimesters (Supplementary Fig. 1). Hair cortisol levels and the prevalence of indicators of recent stress did not differ significantly across trimesters (Supplementary Table 6). Additionally, when analyses were restricted to participants with hair samples collected during the second trimester (n = 87; Supplementary Table 7), estimates were attenuated. Childhood maltreatment was not significantly associated with hair cortisol. Among indicators of recent stress, depressive symptoms, anxiety symptoms, perceived stress, insomnia, and life events in the past month, none showed significant associations with hair cortisol. However, life events in the past year remained marginally associated with lower hair cortisol in the second trimester subset (β = −0.162, 95% CI −0.323 to −0.001, p = 0.048), consistent with primary findings. Of the 87 participants whose hair was collected during the second trimester, 34 (39%) had an estimated four-week hair cortisol concentration window that potentially extended into the first trimester. In the more stringent analysis restricted to participants whose estimated hair cortisol concentration window fell entirely within the second trimester (n = 53), the association between past-year stressful life events and hair cortisol concentrations remained negative and similar in magnitude to the primary estimate, but the confidence interval included zero and the association did not meet the conventional p < 0.05 threshold (B = −0.159, 95% CI: −0.323 to 0.005, p = 0.058; Supplementary Table 8). Finally, when excluding nine participants with hair cortisol concentration values below the assay limit of detection, the estimated association remained similar in direction and magnitude but provided weaker statistical evidence (B = −0.117, 95% CI: −0.234 to 0.000, standardized β = −0.168, p = 0.05; n = 139; Supplementary Table 1). The associations for the other stress-related predictors remained non-significant. Estimates from these analyses were compared with those from the original models and are reported in Supplementary Table 1.

## 5 Discussion

In this study of 148 pregnant women, we found no evidence that childhood maltreatment was associated with hair cortisol levels, either overall or across specific maltreatment subtypes. Among indicators of recent stress, past-year stressful life events were the only factor associated with hair cortisol levels, with higher event counts linked to lower cortisol. An exploratory analysis suggested that high insomnia symptoms may modify the association between childhood maltreatment and cortisol; however, given the exploratory and post-hoc nature of this analysis, findings should be interpreted with caution and require replication in larger independent samples.

The literature on the association between childhood maltreatment and hair cortisol levels during pregnancy shows mixed results, with studies reporting both elevated [23], [59] and reduced cortisol levels [30], [72]. For example, Kelsall and colleagues (n = 1822 pregnant Peruvian women) reported a modest 0.12 log-unit increase in hair cortisol levels among those with a history of childhood abuse [23]. Consistent with our findings, they and others reported no differences when examining specific maltreatment subtypes [23], [73]. Several factors may explain our null findings. The heterogeneity in existing literature likely reflects true variation across samples in maltreatment type, severity, and timing, as well as differences in cortisol measurement approaches and maltreatment operationalization [17], [74], [75]. Furthermore, our sample consisted of predominantly white professional women with relatively low levels of childhood maltreatment exposure, which may have limited statistical power to detect small associations. Finally, individuals with childhood maltreatment histories may exhibit heterogeneous cortisol profiles — ranging from hyperactivation to blunting — that may cancel out at the group level [16], [17], [22]. Notably, different types of maltreatment frequently co-occur [76], as reflected by high intercorrelations among CTQ subscales observed in our sample, complicating efforts to isolate the independent contribution of any single maltreatment type to HPA axis functioning.

A higher number of types of past-year stressful life events was associated with lower hair cortisol levels (p = 0.04); however, the temporal interpretation of the hair cortisol concentration measurement and the sensitivity analyses warrant caution. When the analysis was restricted to participants whose approximate four-week hair cortisol concentration window fell entirely within the second trimester, the estimated association remained negative and similar in magnitude but was less precise and did not meet the conventional significance threshold (p = 0.058). Thus, this sensitivity analysis was directionally consistent with the primary result but did not reach statistical significance. Overall, the finding should therefore be considered exploratory and interpreted cautiously, given the examination of multiple recent-stress indicators, the modest effect size, and the sample size. Replication in larger independent samples is therefore needed. Although we assessed life events occurring anytime in the past year, some may have occurred repeatedly across weeks or months; the observed association may therefore reflect cumulative effects of chronic stress on basal cortisol output rather than responses to discrete acute stressors. In contrast, no significant associations between depression, anxiety, perceived stress, or insomnia and hair cortisol levels were observed. This may reflect the transient or fluctuating nature of these symptoms, which may not produce detectable alterations in longer-term cumulative cortisol exposure as measured by hair cortisol. Additionally, given that our sample consisted of professional women working in healthcare, symptom severity may have been insufficient to produce meaningful alterations in basal cortisol secretion.

Given the exploratory and post-hoc nature of these analyses, the observed interaction between childhood maltreatment and insomnia symptoms should be interpreted cautiously and require replication in larger independent samples. In our exploratory analyses, the combination of moderate-to-severe childhood maltreatment and high insomnia symptoms was associated with lower hair cortisol levels during pregnancy. This direction of association — lower rather than higher cortisol — is consistent with the HPA axis blunting hypothesis, whereby chronic and cumulative adversity exposure, encompassing both early-life maltreatment and persistent sleep disturbance, may be associated with down-regulation of basal cortisol output over time [16], [24], [77]. Insomnia is common during pregnancy due to hormonal and physical changes [78]. In this study, insomnia was operationalized using the WHIIRS as a measure of insomnia symptoms during the past month, with the top 20% (score ≥12) used to identify women with the highest symptom burden given the absence of a validated clinical cutoff. This operationalization captures insomnia symptoms rather than a clinical diagnosis and may reflect a combination of normative pregnancy-related sleep changes and clinically significant sleep disturbance. In the literature, childhood maltreatment has been consistently associated with poor sleep outcomes across the lifespan, including insomnia [79]. For example, among 634 pregnant and early postpartum women, those with histories of childhood physical or sexual abuse had 1.7 times higher odds of sleep disturbance and 2.1 times higher odds of poor sleep quality compared to women without such histories, with risk increasing for those experiencing multiple forms of abuse [80]. The lower cortisol levels observed among maltreated women with high insomnia symptoms may be relevant in the pregnancy context, given the critical role of cortisol in fetal organ maturation and neurodevelopment; however, the direction and clinical significance of this association remain uncertain. Prior studies have documented associations between pregnancy insomnia and adverse maternal outcomes, including gestational diabetes and perinatal depression, as well as adverse child outcomes [78], [81]. However, whether HPA axis dysregulation underlies these associations warrants further investigation.

Several limitations should be noted. First, participants were predominantly white professionals working in healthcare, characterized as a highly demanding occupation, and thus may be exposed to high occupational stress (e.g., shift work, long hours, high workload) impacting both functioning of the HPA-axis and sleep quality. As a result, findings may not generalize to the broader pregnant population. Second, this study did not include data on the number of children or partnership status, both of which may influence stress levels and cortisol during pregnancy and may represent sources of residual confounding. Third, our sample had relatively low levels of childhood maltreatment compared with population estimates; future studies should examine these questions in more racially, ethnically, and socioeconomically diverse samples, and in cohorts with abuse exposure distributions that more closely reflect those of the general population. Fourth, the 3-cm hair segment reflects approximately three months of cumulative cortisol secretion; given a mean gestational age of 18.3 weeks at sampling (range: 9–33 weeks), for some participants this window extends into the preconception period, introducing potential misclassification of gestational cortisol exposure. Fifth, retrospective self-report of childhood maltreatment may introduce recall bias. Sixth, some psychological distress scales evaluate shorter time frames, such as the last week or last two weeks, compared to the three months represented by 3 cm hair segments, potentially leading to a mismatch between predictor and outcome measurement. Seventh, the cross-sectional and observational nature of this study precludes conclusions about the directionality of observed associations; for example, lower basal cortisol may precede rather than follow the development of insomnia symptoms. Finally, while Bonferroni correction was applied to secondary analyses. We did not correct the exploratory insomnia interaction for multiple testing, as it was specified post hoc and tested in a limited subset of models. This result should therefore be viewed as hypothesis-generating and interpreted with caution.

Nonetheless, despite these limitations, this study includes a sample of predominantly second-trimester pregnant women to capture cortisol dynamics during a critical period of fetal development; use of hair cortisol to capture cumulative cortisol exposure over several months; validated instruments for assessing childhood maltreatment (including abuse and neglect subscales) and recent stress indicators with hair cortisol.

In conclusion, among 148 pregnant women from Nurses’ Health Study 3, childhood maltreatment was not associated with hair cortisol levels, either overall or specific maltreatment subtypes. Past-year stressful life events were associated with lower hair cortisol levels, suggesting a possible link between cumulative stress exposure and blunted HPA axis activity during pregnancy. An exploratory finding suggested that insomnia symptoms may modify the association between childhood maltreatment and cortisol levels, though this requires replication in larger independent samples. Future prospective studies in larger and more diverse samples are needed to clarify the associations between childhood maltreatment, sleep disturbance, and HPA axis functioning during pregnancy. Longitudinal designs tracking cortisol across trimesters would help clarify whether these associations vary by gestational timing. The exploratory finding regarding insomnia as a potential moderator warrants targeted investigation, including examination of whether sleep disturbance represents a modifiable pathway linking childhood maltreatment to HPA axis dysregulation during pregnancy, and the downstream implications for maternal and offspring health.

## CRediT authorship contribution statement

**Michelle M.J. Mens:** Writing – review & editing, Writing – original draft, Methodology, Investigation, Formal analysis, Data curation, Conceptualization. **Chirag M. Vyas:** Writing – review & editing, Methodology, Investigation, Formal analysis, Data curation. **Bohao Wu:** Writing – review & editing, Investigation, Formal analysis, Data curation. **Natalie Slopen:** Writing – review & editing, Methodology, Investigation. **Shruthi Mahalingaiah:** Writing – review & editing, Methodology, Funding acquisition. **Jorge E. Chavarro:** Writing – review & editing, Project administration, Investigation, Funding acquisition, Conceptualization. **Rajarshi Mukherjee:** Writing – review & editing, Funding acquisition, Formal analysis, Conceptualization. **Marc G. Weisskopf:** Writing – review & editing, Funding acquisition, Conceptualization. **Andrea L. Roberts:** Writing – review & editing, Supervision, Project administration, Methodology, Investigation, Funding acquisition, Formal analysis, Conceptualization.

## Data availability statement

Access to de-identified data, data dictionaries to code used to generate study data will be granted within the study's data repository system after obtaining authorization from our local IRB and putting in place Data Use Agreements with the parties interested in accessing study data Further information including the procedures to obtain and access data from the Nurses' Health Studies is described at https://www.nurseshealthstudy.org/researchers (contact email: nhsaccess@channing.harvard.edu).

## Role of the funder/sponsor

The funders had no role in the design and conduct of the study; collection, management, analysis, and interpretation of the data; preparation, review, or approval of the manuscript; and decision to submit the manuscript for publication.

## Declaration of competing interest

Dr. Vyas has received research support from Alzheimer's Association, Mars Edge, and American Foundation for Suicide Prevention during the conduct of the study. He has also received honoraria from Haleon.

Dr. Chavarro has received grants from the NIH during the conduct of the study and grants from the National Institute of Health (NIH), US Food and Drug Administration, and Centers for Disease Control and Prevention outside of the submitted work.

Dr. Roberts has received funding from the Dean's Acceleration Award from Harvard T.H. Chan School of Public Health, the Broad Institute, and the NIH during the conduct of the study.

No other disclosures were reported.
