# Supplementary material for: Childhood maltreatment and hair cortisol in pregnancy: role of recent stress
Source: Compr Psychoneuroendocrinol. 2026 Nov 2;28:100389. doi: 10.1016/j.cpnec.2026.100389 (PMC13760102; doi:10.1016/j.cpnec.2026.100389)
Supplement: Supplementary file 1 [file mmc1.docx]

**Online Supplement**

**Supplementary Figure 1. Histogram of log hair cortisol across trimesters**

Density of log hair cortisol levels per trimester, including first trimester (blue), second trimester (green), third trimester (red). Illustrated is that the mean values are increasing when pregnancy progresses. Majority of participants are in the second trimester (58.8%), followed by the first trimester (29.1%), and third trimester (12.2%).

# Supplementary Table 1. Sensitivity analysis comparing original estimates with estimates excluding hair cortisol concentration values below the limit of detection

| Predictor | Analysis | N | B | SE | β | 95% CI | p |
| --- | --- | --- | --- | --- | --- | --- | --- |
| Childhood maltreatment (CTQ total score) | Original analysis | 148 | 0.003 | 0.008 | 0.028 | -0.014, 0.019 | 0.742 |
| Childhood maltreatment (CTQ total score) | Excluding below-LOD | 139 | -0.005 | 0.008 | -0.052 | -0.020, 0.010 | 0.548 |
| Depressive symptoms (CES-D) | Original analysis | 148 | 0.007 | 0.022 | 0.024 | -0.038, 0.051 | 0.770 |
| Depressive symptoms (CES-D) | Excluding below-LOD | 139 | 0.007 | 0.020 | 0.028 | -0.034, 0.047 | 0.743 |
| Anxiety symptoms (GAD) | Original analysis | 148 | -0.053 | 0.078 | -0.057 | -0.206, 0.100 | 0.496 |
| Anxiety symptoms (GAD) | Excluding below-LOD | 139 | -0.071 | 0.071 | -0.086 | -0.211, 0.069 | 0.315 |
| Perceived stress (PSS) | Original analysis | 148 | 0.014 | 0.035 | 0.035 | -0.054, 0.083 | 0.680 |
| Perceived stress (PSS) | Excluding below-LOD | 139 | 0.002 | 0.032 | 0.006 | -0.060, 0.065 | 0.943 |
| Life events, past month (LEDS) | Original analysis | 148 | 0.010 | 0.171 | 0.005 | -0.328, 0.347 | 0.954 |
| Life events, past month (LEDS) | Excluding below-LOD | 139 | -0.074 | 0.152 | -0.042 | -0.374, 0.227 | 0.629 |
| Life events, past year (LEDS) | Original analysis | 148 | -0.135 | 0.065 | -0.172 | -0.263, -0.006 | 0.040 |
| Life events, past year (LEDS) | Excluding below-LOD | 139 | -0.117 | 0.059 | -0.168 | -0.234, 0.000 | 0.050 |
| Insomnia symptoms (WHIIRS) | Original analysis | 148 | 0.001 | 0.020 | 0.004 | -0.038, 0.040 | 0.960 |
| Insomnia symptoms (WHIIRS) | Excluding below-LOD | 139 | -0.008 | 0.018 | -0.039 | -0.043, 0.027 | 0.654 |

**Note.** B = unstandardized regression coefficient; β = standardized regression coefficient; SE = standard error; CI = confidence interval; LOD = limit of detection; CTQ = Childhood Trauma Questionnaire; CES-D = Center for Epidemiological Studies Depression Scale; GAD = Generalized Anxiety Disorder scale; PSS = Perceived Stress Scale; LEDS = Life Events and Difficulties Schedule; WHIIRS = Women’s Health Initiative Insomnia Rating Scale. Estimates are from separate multivariable linear regression models adjusted for assay batch, gestational age at hair sampling (weeks), exercise/swimming, and topical, oral, or nasal cortisol use. “Original analysis” includes all participants, with hair cortisol values below the LOD substituted according to the laboratory protocol; “Excluding below-LOD” excludes the nine participants with hair cortisol values below the assay LOD of 0.45 pg/mg. Standardized coefficients are provided to facilitate comparison of effect sizes across continuous predictors measured on different scales.

**Supplementary Table 2. Log-transformed hair cortisol levels by childhood maltreatment severity group**

| Childhood maltreatment severity group | n | Mean (SD) | Median (IQR) |
| --- | --- | --- | --- |
| No maltreatment | 28 | 0.89 (1.28) | 0.95 (1.42) |
| Very low | 42 | 1.03 (0.82) | 0.96 (0.76) |
| Low | 43 | 1.14 (1.12) | 0.96 (0.84) |
| Moderate–severe | 35 | 1.06 (0.75) | 1.10 (1.05) |

**Note**. SD = standard deviation; IQR = interquartile range. Childhood maltreatment severity was based on the Childhood Trauma Questionnaire (CTQ) total score and categorized as no maltreatment (CTQ = 25), very low maltreatment (CTQ = 26–29), low maltreatment (CTQ = 30–36), and moderate-to-severe maltreatment (CTQ > 36). Hair cortisol concentrations were log-transformed prior to calculation of the descriptive statistics.

# Supplementary Table 3. Complete estimates from primary regression models

| Model | term | B | SE | β | 95% CI | p |
| --- | --- | --- | --- | --- | --- | --- |
| Childhood maltreatment (CTQ total) | (Intercept) | 0.673 | 0.394 |  | -0.105, 1.451 | 0.089 |
| Childhood maltreatment (CTQ total) | Childhood maltreatment score | 0.003 | 0.008 | 0.028 | -0.014, 0.019 | 0.742 |
| Childhood maltreatment (CTQ total) | Gestational age (weeks) | 0.016 | 0.013 | 0.105 | -0.010, 0.043 | 0.214 |
| Childhood maltreatment (CTQ total) | Batch | -0.036 | 0.170 | -0.018 | -0.372, 0.300 | 0.833 |
| Childhood maltreatment (CTQ total) | Exercise/swimming | 0.225 | 0.287 | 0.067 | -0.343, 0.793 | 0.436 |
| Childhood maltreatment (CTQ total) | Topical/oral cortisol | 0.063 | 0.314 | 0.017 | -0.558, 0.684 | 0.842 |
| Childhood maltreatment (CTQ total) | Nasal cortisol | -0.287 | 0.267 | -0.090 | -0.815, 0.240 | 0.283 |
| Depressive symptoms (CES-D) | (Intercept) | 0.730 | 0.306 |  | 0.126, 1.334 | 0.018 |
| Depressive symptoms (CES-D) | Depressive score | 0.007 | 0.022 | 0.024 | -0.038, 0.051 | 0.770 |
| Depressive symptoms (CES-D) | Gestational age (weeks) | 0.016 | 0.013 | 0.104 | -0.010, 0.042 | 0.221 |
| Depressive symptoms (CES-D) | Batch | -0.033 | 0.170 | -0.016 | -0.368, 0.302 | 0.847 |
| Depressive symptoms (CES-D) | Exercise/swimming | 0.227 | 0.287 | 0.067 | -0.341, 0.795 | 0.430 |
| Depressive symptoms (CES-D) | Topical/oral cortisol | 0.056 | 0.314 | 0.015 | -0.565, 0.677 | 0.858 |
| Depressive symptoms (CES-D) | Nasal cortisol | -0.292 | 0.266 | -0.092 | -0.819, 0.235 | 0.275 |
| Anxiety symptoms (GAD) | (Intercept) | 0.829 | 0.297 |  | 0.241, 1.417 | 0.006 |
| Anxiety symptoms (GAD) | Anxiety score | -0.053 | 0.078 | -0.057 | -0.206, 0.100 | 0.496 |
| Anxiety symptoms (GAD) | Gestational age (weeks) | 0.016 | 0.013 | 0.102 | -0.010, 0.042 | 0.226 |
| Anxiety symptoms (GAD) | Batch | -0.035 | 0.169 | -0.018 | -0.370, 0.299 | 0.834 |
| Anxiety symptoms (GAD) | Exercise/swimming | 0.222 | 0.287 | 0.066 | -0.345, 0.789 | 0.440 |
| Anxiety symptoms (GAD) | Topical/oral cortisol | 0.042 | 0.315 | 0.011 | -0.579, 0.664 | 0.893 |
| Anxiety symptoms (GAD) | Nasal cortisol | -0.290 | 0.266 | -0.091 | -0.816, 0.236 | 0.278 |
| Perceived stress (PSS) | (Intercept) | 0.723 | 0.299 |  | 0.131, 1.314 | 0.017 |
| Perceived stress (PSS) | Perceived stress score | 0.014 | 0.035 | 0.035 | -0.054, 0.083 | 0.680 |
| Perceived stress (PSS) | Gestational age (weeks) | 0.016 | 0.013 | 0.102 | -0.010, 0.042 | 0.228 |
| Perceived stress (PSS) | Batch | -0.030 | 0.170 | -0.015 | -0.365, 0.306 | 0.862 |
| Perceived stress (PSS) | Exercise/swimming | 0.211 | 0.291 | 0.062 | -0.364, 0.785 | 0.469 |
| Perceived stress (PSS) | Topical/oral cortisol | 0.077 | 0.317 | 0.020 | -0.549, 0.703 | 0.809 |
| Perceived stress (PSS) | Nasal cortisol | -0.289 | 0.267 | -0.091 | -0.816, 0.238 | 0.281 |
| Life events, past month (LEDS) | (Intercept) | 0.763 | 0.283 |  | 0.205, 1.322 | 0.008 |
| Life events, past month (LEDS) | Life event score, month | 0.010 | 0.171 | 0.005 | -0.328, 0.347 | 0.954 |
| Life events, past month (LEDS) | Gestational age (weeks) | 0.016 | 0.013 | 0.104 | -0.010, 0.043 | 0.222 |
| Life events, past month (LEDS) | Batch | -0.033 | 0.170 | -0.016 | -0.368, 0.303 | 0.848 |
| Life events, past month (LEDS) | Exercise/swimming | 0.228 | 0.289 | 0.067 | -0.343, 0.799 | 0.432 |
| Life events, past month (LEDS) | Topical/oral cortisol | 0.059 | 0.315 | 0.016 | -0.563, 0.680 | 0.852 |
| Life events, past month (LEDS) | Nasal cortisol | -0.293 | 0.267 | -0.092 | -0.820, 0.234 | 0.274 |
| Life events, past year (LEDS) | (Intercept) | 0.936 | 0.290 |  | 0.363, 1.510 | 0.002 |
| Life events, past year (LEDS) | Life events score, year | -0.135 | 0.065 | -0.172 | -0.263, -0.006 | 0.040 |
| Life events, past year (LEDS) | Gestational age (weeks) | 0.018 | 0.013 | 0.116 | -0.008, 0.044 | 0.164 |
| Life events, past year (LEDS) | Batch | -0.057 | 0.168 | -0.029 | -0.389, 0.274 | 0.733 |
| Life events, past year (LEDS) | Exercise/swimming | 0.227 | 0.283 | 0.067 | -0.332, 0.786 | 0.423 |
| Life events, past year (LEDS) | Topical/oral cortisol | -0.018 | 0.312 | -0.005 | -0.634, 0.598 | 0.955 |
| Life events, past year (LEDS) | Nasal cortisol | -0.292 | 0.263 | -0.092 | -0.811, 0.227 | 0.268 |
| Insomnia symptoms (WHIIRS) | (Intercept) | 0.758 | 0.307 |  | 0.152, 1.364 | 0.015 |
| Insomnia symptoms (WHIIRS) | Insomnia score | 0.001 | 0.020 | 0.004 | -0.038, 0.040 | 0.960 |
| Insomnia symptoms (WHIIRS) | Gestational age (weeks) | 0.016 | 0.013 | 0.104 | -0.010, 0.043 | 0.223 |
| Insomnia symptoms (WHIIRS) | Batch | -0.033 | 0.170 | -0.017 | -0.369, 0.302 | 0.845 |
| Insomnia symptoms (WHIIRS) | Exercise/swimming | 0.229 | 0.288 | 0.068 | -0.341, 0.798 | 0.428 |
| Insomnia symptoms (WHIIRS) | Topical/oral cortisol | 0.061 | 0.316 | 0.016 | -0.563, 0.686 | 0.846 |
| Insomnia symptoms (WHIIRS) | Nasal cortisol | -0.293 | 0.267 | -0.092 | -0.822, 0.235 | 0.274 |

Note. B = unstandardized regression coefficient; β = standardized regression coefficient; SE = standard error; CI = confidence interval; CTQ = Childhood Trauma Questionnaire; CES-D = Center for Epidemiological Studies Depression Scale; GAD = Generalized Anxiety Disorder scale; PSS = Perceived Stress Scale; LEDS = Life Events and Difficulties Schedule; WHIIRS = Women’s Health Initiative Insomnia Rating Scale. Each block presents estimates from a separate multivariable linear regression model with log-transformed hair cortisol concentration as the outcome and the indicated childhood maltreatment or recent-stress measure as the focal predictor. All models were adjusted for gestational age at hair sampling (weeks), assay batch, exercise/swimming, topical/oral corticosteroid use, and nasal corticosteroid use. B represents the expected change in log-transformed hair cortisol associated with a one-unit increase in a continuous predictor or with the indicated category relative to its reference category. Standardized β values are provided to facilitate comparison of effect sizes across predictors measured on different scales; standardized coefficients are not reported for the intercept. Covariate estimates are presented for transparency and reproducibility and were not interpreted as independent study findings.

**Supplementary Table 4. Association between types of childhood maltreatment and log-transformed hair cortisol levels.**

| Type of childhood maltreatment | B | β | SE | 95% CI | *p* |
| --- | --- | --- | --- | --- | --- |
| Emotional abuse | 0.019 | 0.068 | 0.024 | −0.028, 0.066 | 0.423 |
| Physical abuse | 0.007 | 0.010 | 0.063 | −0.118, 0.132 | 0.909 |
| Sexual abuse | −0.019 | −0.055 | 0.029 | −0.077, 0.039 | 0.513 |
| Emotional neglect | 0.024 | 0.081 | 0.024 | −0.024, 0.072 | 0.334 |
| Physical neglect | −0.029 | −0.053 | 0.046 | −0.120, 0.062 | 0.528 |

**Note.** B = unstandardized regression coefficient; β = standardized regression coefficient; SE = standard error; CI = confidence interval; CTQ = Childhood Trauma Questionnaire. Each maltreatment subtype was examined as a continuous predictor in a separate multivariable linear regression model with log-transformed hair cortisol concentration as the outcome. All models were adjusted for gestational age at hair sampling (weeks), assay batch, exercise/swimming, and topical, oral, or nasal cortisol use. B represents the expected change in log-transformed hair cortisol associated with a one-unit increase in the corresponding CTQ subscale score. Standardized β values are provided to facilitate comparison of effect sizes across maltreatment subtypes. Bonferroni correction was applied across the five subtype analyses, with a corrected significance threshold of *p* < 0.010.

**Supplementary Table 5. Effect modification by recent stress in the association between childhood maltreatment and log-transformed hair cortisol levels.**

| Effect modifier (recent stress indicator; continuous) | B (interaction) | SE | 95% CI | p-value |
| --- | --- | --- | --- | --- |
| CTQ × depressive symptoms | -0.002 | 0.003 | -0.007, 0.003 | 0.400 |
| CTQ × anxiety symptoms | -0.007 | 0.010 | -0.026, 0.013 | 0.489 |
| CTQ × perceived stress | 0.001 | 0.003 | -0.006, 0.008 | 0.738 |
| CTQ × life events, past month | -0.006 | 0.018 | -0.040, 0.029 | 0.755 |
| CTQ × life events, past year | -0.001 | 0.006 | -0.013, 0.010 | 0.834 |
| CTQ × insomnia symptoms | -0.003 | 0.003 | -0.009, 0.002 | 0.219 |

**Note.** B = unstandardized regression coefficient; SE = standard error; CI = confidence interval; CTQ = Childhood Trauma Questionnaire. B (interaction) represents the unstandardized regression coefficient for the product term between the continuous CTQ total score and each continuous recent-stress indicator. Each interaction was estimated in a separate multivariable linear regression model with log-transformed hair cortisol concentration as the outcome and included the corresponding main effects and interaction term. All models were adjusted for gestational age at hair sampling (weeks), assay batch, exercise/swimming, and topical, oral, or nasal cortisol use. Continuous predictors were entered in their original measurement units and were not z-standardized before calculation of the interaction terms. Statistical significance was evaluated using the interaction coefficient, 95% confidence interval, and *p*-value. Bonferroni correction was applied across the interaction models, with a corrected significance threshold of *p* < 0.010.

**Supplementary Table 6. Hair cortisol levels and distribution of recent stress indicators, stratified by trimester of pregnancy.**

|  | First trimester (n=43) | Second trimester (n=87) | Third trimester (n=18) | p-value |
| --- | --- | --- | --- | --- |
| log hair cortisol (mean (SD)) | 0.84 (0.90) | 1.09 (0.96) | 1.25 (1.29) | 0.25 |
| Perceived stress continuous (mean (SD)) | 3.16 (2.35) | 3.29 (2.60) | 3.83 (1.95) | 0.62 |
| High perceived stress (%) | 6 (14.0) | 18 (20.7) | 4 (22.2) | 0.61 |
| Depressive symptoms continuous (mean (SD)) | 5.28 (3.76) | 6.03 (3.71) | 6.56 (3.31) | 0.39 |
| High depression (%) | 5 (11.6) | 17 (19.5) | 3 (16.7) | 0.53 |
| Anxiety continuous (mean (SD)) | 0.95 (1.09) | 1.03 (1.09) | 1.17 (0.92) | 0.78 |
| High anxiety (%) | 5 (11.6) | 7 (8.0) | 1 (5.6) | 0.70 |
| Stressful life events month (%) |  |  |  | 0.68 |
| 0 | 33 (76.7) | 64 (73.6) | 13 (72.2) |  |
| 1 | 10 (23.3) | 20 (23.0) | 5 (27.8) |  |
| 2 | 0 (0.0) | 3 (3.4) | 0 (0.0) |  |
| Stressful life events year (%) |  |  |  | 0.85 |
| 0 | 13 (30.2) | 24 (27.6) | 3 (16.7) |  |
| 1 | 15 (34.9) | 33 (37.9) | 8 (44.4) |  |
| 2 | 8 (18.6) | 13 (14.9) | 3 (16.7) |  |
| 3 | 5 (11.6) | 12 (13.8) | 2 (11.1) |  |
| 4 | 2 (4.7) | 1 (1.1) | 1 (5.6) |  |
| 5 | 0 (0.0) | 4 (4.6) | 1 (5.6) |  |
| Insomnia continuous (mean (SD)) | 7.65 (4.09) | 8.28 (4.36) | 9.83 (4.44) | 0.20 |
| High insomnia (%) | 6 (14.0) | 14 (16.1) | 7 (38.9) | 0.05 |

**Note.** Values are presented as mean (SD) for continuous variables and *n* (%) for categorical variables. SD = standard deviation. High perceived stress was defined as PSS-4 ≥ 6; high depressive symptoms as CES-D-10 ≥ 10; high anxiety symptoms as GAD-2 ≥ 3; and high insomnia symptoms as WHIIRS ≥ 12 (top 20% of the distribution). Stressful life events represent the number of events reported during the past month or past year. *p*-values represent comparisons across the three trimesters.

**Supplementary Table 7. Association between childhood maltreatment, indicators of recent stress, and hair cortisol levels among women in their second trimester of pregnancy (n = 87).**

| Predictor | B | SE | β | 95% CI | p |
| --- | --- | --- | --- | --- | --- |
| Childhood maltreatment (CTQ total score) | 0.002 | 0.011 | 0.024 | -0.020, 0.025 | 0.828 |
| Depressive symptoms (CES-D) | -0.026 | 0.029 | -0.102 | -0.084, 0.031 | 0.362 |
| Anxiety symptoms (GAD) | -0.117 | 0.100 | -0.133 | -0.317, 0.082 | 0.246 |
| Perceived stress (PSS) | -0.008 | 0.042 | -0.021 | -0.092, 0.077 | 0.855 |
| Life events, past month (LEDS) | -0.084 | 0.206 | -0.046 | -0.493, 0.325 | 0.685 |
| Life events, past year (LEDS) | -0.162 | 0.081 | -0.220 | -0.323, -0.001 | 0.048 |
| Insomnia symptoms (WHIIRS) | -0.019 | 0.025 | -0.085 | -0.068, 0.031 | 0.451 |

**Note.** B = unstandardized regression coefficient; β = standardized regression coefficient; SE = standard error; CI = confidence interval; CTQ = Childhood Trauma Questionnaire; CES-D = Center for Epidemiological Studies Depression Scale; GAD = Generalized Anxiety Disorder scale; PSS = Perceived Stress Scale; LEDS = Life Events and Difficulties Schedule; WHIIRS = Women’s Health Initiative Insomnia Rating Scale. Analyses were restricted to participants whose hair samples were collected during the second trimester (*n* = 87). Each predictor was examined separately in a multivariable linear regression model with log-transformed hair cortisol concentration as the outcome. All models were adjusted for gestational age at hair sampling (weeks), assay batch, exercise/swimming, and topical, oral, or nasal cortisol use. B represents the expected change in log-transformed hair cortisol associated with a one-unit increase in the corresponding predictor. Standardized β values are provided to facilitate comparison of effect sizes across continuous predictors measured on different scales.

# Supplementary Table 8. Sensitivity analysis: entire approximate 4-week hair window in second trimester

| Predictor | B | SE | β | 95% CI | p |
| --- | --- | --- | --- | --- | --- |
| Childhood maltreatment (CTQ total score) | -0.004 | 0.011 | -0.050 | -0.025, 0.018 | 0.739 |
| Depressive symptoms (CES-D) | -0.052 | 0.031 | -0.246 | -0.113, 0.010 | 0.099 |
| Anxiety symptoms (GAD) | -0.163 | 0.106 | -0.227 | -0.376, 0.050 | 0.131 |
| Perceived stress (PSS) | -0.085 | 0.045 | -0.287 | -0.175, 0.005 | 0.065 |
| Life events, past month (LEDS) | 0.007 | 0.201 | 0.006 | -0.397, 0.411 | 0.971 |
| Life events, past year (LEDS) | -0.159 | 0.082 | -0.283 | -0.323, 0.005 | 0.058 |
| Insomnia symptoms (WHIIRS) | -0.015 | 0.026 | -0.083 | -0.066, 0.037 | 0.571 |

**Note.** B = unstandardized regression coefficient; β = standardized regression coefficient; SE = standard error; CI = confidence interval; CTQ = Childhood Trauma Questionnaire; CES-D = Center for Epidemiological Studies Depression Scale; GAD = Generalized Anxiety Disorder scale; PSS = Perceived Stress Scale; LEDS = Life Events and Difficulties Schedule; WHIIRS = Women’s Health Initiative Insomnia Rating Scale. Analyses were restricted to participants whose estimated four-week hair cortisol level exposure window fell entirely within the second trimester (*n* = 53). Assuming that 1 cm of hair represents approximately four weeks of cortisol accumulation, the beginning of the hair cortisol level exposure window was estimated by subtracting four weeks from gestational age at hair collection; this corresponded to hair collection at gestational weeks 18–27. Each predictor was examined separately in a multivariable linear regression model with log-transformed hair cortisol concentration as the outcome. All models were adjusted for gestational age at hair sampling (weeks), assay batch, exercise/swimming, and topical, oral, or nasal cortisol use. B represents the expected change in log-transformed hair cortisol associated with a one-unit increase in the corresponding predictor. Standardized β values are provided to facilitate comparison of effect sizes across continuous predictors measured on different scales.

**Supplementary Methods**

**Covariate assessment and selection**

The selection of covariates was based on previous literature (Gerber et al., 2012; Orta et al., 2019; Schreier et al., 2015; Stalder et al., 2017). Participants’ birthdate and racial identity (White, Black, Asian, Hispanic, Other) was self-reported on the NHS3 baseline questionnaire. When the participant agreed to provide a hair sample, we sent a questionnaire to obtain gestational age, use of hair treatment (coloring, bleaching, straightening), hair washing in the last 24 hours, typical exercise>2h/day, swimming in chorine pool (≥1/week for last 3 months), cortisol medication use (topical, oral, or nasal), medical conditions and their medication use, including diabetes mellitus, thyroid disorders (e.g., hypothyroidism or hyperthyroidism), and other dysregulations of the HPA axis (e.g., Cushing's disease or Addison's disease). Participants’ age at hair collection was calculated in years between date of hair collection and participants’ birthdate. Before including these nuisance variables in the models to reduce unexplained variance, we first assessed their association with hair cortisol levels. We compared mean hair cortisol levels using a t-test for categorical variables and regression analysis for continuous variables. Hair cortisol concentrations were not associated with: hair washing in the last 24 hours (p=0.56), bleached hair (p = 0.80), colored hair (p = 0.87), straightened hair (p = 0.71), medical conditions, or medication use (p = 0.36), and we therefore did not include these as covariates. Also, age was not a significant nuance variable (p=0.30) and was not used in the statistical analysis. Additionally, given that our sample was predominantly White (n = 133; 89.5%), we tested for differences in hair cortisol across racial identity groups (Asian: n=3 [2.0%], Black: n = 3 [2.0%], Hispanic: n=1 [0.7%], Other: n=8 [5.4%]) using an ANOVA followed by Tukey's Honestly Significant Difference post hoc test. No significant racial differences in hair cortisol concentrations were observed **(**all adjusted p-values >0.05), therefore racial identity was not included as covariate. Given the low prevalence of oral cortisol users (n=2), and the similar associations of topical and oral steroid use, those were included as a combined variable. Similarly, the prevalence of typical exercise >2h/day was low (n=1) and was therefore combined with swimming.

**References**

Gerber, M., Brand, S., Lindwall, M., Elliot, C., Kalak, N., Herrmann, C., Pühse, U., Jonsdottir, I.H., 2012. Concerns regarding hair cortisol as a biomarker of chronic stress in exercise and sport science. J Sports Sci Med 11, 571-581.

Orta, O.R., Tworoger, S.S., Terry, K.L., Coull, B.A., Gelaye, B., Kirschbaum, C., Sanchez, S.E., Williams, M.A., 2019. Stress and hair cortisol concentrations from preconception to the third trimester. Stress 22, 60-69.

Schreier, H.M., Enlow, M.B., Ritz, T., Gennings, C., Wright, R.J., 2015. Childhood abuse is associated with increased hair cortisol levels among urban pregnant women. J Epidemiol Community Health 69, 1169-1174.

Stalder, T., Steudte-Schmiedgen, S., Alexander, N., Klucken, T., Vater, A., Wichmann, S., Kirschbaum, C., Miller, R., 2017. Stress-related and basic determinants of hair cortisol in humans: A meta-analysis. Psychoneuroendocrinology 77, 261-274.
